# Supplementary material for: Are carbon emissions trading and green financial instruments synergistic? -Comprehensive quantitative research based on content analysis
Source: PLoS One. 2024 Mar 7;19(3):e0298601. doi: 10.1371/journal.pone.0298601 (PMC10919601; doi:10.1371/journal.pone.0298601)
Supplement: S1 Data — (ZIP) [file pone.0298601.s001.zip › Data/地级市Z_F工作报告.zip!/╕╩╦α/╬Σ═■╩╨/└┤╫╘╣1⁄2╓┌║┼í╛┬φ┐╦╩2╛▌═°í┐_2018.docx]

各位代表：

　　我代表市人民政府向大会作工作报告，请予审议，并请市政协委员和列席同志提出意见。

2017年主要工作

　　今年以来，在省委、省政府和市委的坚强领导下，市政府深入贯彻党的十八大和十八届三中、四中、五中、六中、七中全会精神，认真学习贯彻党的十九大精神，全面落实习近平总书记视察甘肃重要讲话和“八个着力”重要指示精神，按照省第十三次党代会和市委四届四次五次全会、市四届人大一次会议部署，统筹推进“五位一体”总体布局和协调推进“四个全面”战略布局，坚定不移贯彻“走生态优先、绿色发展之路，努力建设经济强市、生态大市、文化旅游名市，全力打造生态美、产业优、百姓富的和谐武威”的总体思路，坚持稳中求进工作总基调，坚持以供给侧结构性改革为主线，狠抓稳增长、促改革、调结构、惠民生、防风险各项政策措施落实。紧盯脱贫攻坚、全面建成小康社会目标，突出抓重点、补短板、强弱项，全力攻坚、止滑促稳。预计全市实现生产总值442.8亿元，工业增加值69亿元，固定资产投资325.9亿元，社会消费品零售总额194.2亿元，大口径财政收入52.5亿元，一般公共预算收入30.2亿元，城镇居民人均可支配收入25620元，农村居民人均可支配收入10570元，居民消费价格指数为101%。

　　一是精准扶贫精准脱贫进展明显。把脱贫攻坚作为基础性、底线性任务，开展精细精确精微“绣花式”扶贫。预计全年减贫2.93万人，贫困发生率由2016年的6.32%下降到4.29%，民勤县、凉州区可摘帽退出。贫困村新建设施农牧业2.5万亩、特色林果业0.7万亩，发展马铃薯、中药材等特色产业29.5万亩。2016年易地扶贫搬迁入住率为64%，2017年规划新建的3970户易地扶贫搬迁移民住宅主体完工。培训贫困人口10587人，输转劳动力3.35万人，实现劳务收入6.3亿元。新建改建贫困村学校（幼儿园）19所，建设贫困村综合性文化中心13个、饮水安全巩固提升工程10处，建成贫困片（区）农村公路1326公里。加大东西部扶贫协作力度，交通银行、天津市蓟州区等单位对口帮扶古浪县、天祝县工作深入推进。新增精准扶贫专项贷款2.4亿元，投入财政扶贫资金15.7亿元、比上年增长14.9%。加强扶贫项目资金专项检查、专项审计、专项巡查，严肃查处和纠正违纪违规行为。

　　二是现代产业体系加快培育。启动编制武威市和各县区县域经济发展规划，推进产业转型升级。新建设施农牧业3.7万亩，新增规模养殖户1336户；补植补造特色林果23.6万亩，建成天祝藜麦产业基地5000亩。新建冷藏库容7.9万吨、累计达到39.7万吨。推广旱作农业技术71.4万亩，发展高效节水灌溉农业25万亩。预计实现第一产业增加值116.8亿元，增长6%。甘肃武威国家农业科技园区通过科技部验收。推进工业转型升级，开展产业园区功能定位评估论证，制定实施推进生态工业发展、鼓励支持工业企业“规下转规上”、政银险担合作小额贷款风险补偿等政策措施，加快培育高端装备制造、新材料、新能源、生物制药、信息技术、节能环保等战略性新兴产业，强化工业经济运行调度。新增规模以上企业25户。新认定高新技术企业6户，培育省级科技创新型企业7户。1—11月，全市工业用电量增长10.6%，规上工业企业产成品库存下降8.3%、利润增长73.9%、税收增长21.1%。加快推进文化旅游产业融合发展，出台支持文化旅游业发展的若干政策，市文化旅游发展集团组建运行，全域旅游、乡村旅游发展规划完成初稿编制。北京巅峰智业、省公航旅等文化旅游合作项目加快推进。凉州文化论坛暨中国·河西走廊第七届有机葡萄美酒节成功举办。预计全年实现旅游接待人数1183.4万人（次）、增长23%，旅游总收入63.1亿元、增长26.1%。甘肃（武威）国际陆港建设加快推进，进境木材检验检疫集中监管区、进口肉类指定查验场基本建成。“天马号”发运66列，货运量14.7万吨、货运总值1.6亿美元。

　　三是项目建设和招商引资持续推进。制定实施《关于抓好当前“三重”任务落实促进经济趋稳向好的实施方案》《“一业一策、一企一策、一事一议”的实施方案》，开展攻坚作战行动，实行市级领导定向包县、驻点督导，建立项目清单管理、市县区两级调度、实施过程控制机制，狠抓项目前期工作，提高审批效率，全力推动项目落地。实施500万元及以上项目665项，其中亿元以上136项；实施招商引资项目670项，落实到位资金418亿元。PPP项目工作连续两年受到国务院表彰奖励。甘肃正阳马铃薯主食化生产线（一期）、路斯宠物食品生产线（一期）、石岛高档玻璃制品（一期）、凯帝斯智能立体停车场设施设备加工等项目建成投产，天祝宏氟锂业科技锂电池新材料产业园、众兴食用菌循环经济产业园等项目加快推进。万达广场主体完工，国家、省级电子商务示范项目实现县区全覆盖。天（祝）景（泰）二级公路、民勤大滩至南湖公路建成通车，北（山）仙（米寺）高速公路武威至民勤段和凉州至古浪、大靖至干城、高坝至祁连、金色大道至白塔寺景区等公路加快建设。红崖山水库加高扩建工程主体完工，甘电投2×350兆瓦热电联产、亿利洁能微煤雾化热电联产项目建成投产，2017年农网改造升级工程顺利完成。

　　四是全域生态文明建设成效显著。认真贯彻中办、国办和省“两办”《通报》精神，深刻汲取教训，完善中央环保督察反馈意见整改方案和祁连山自然保护区生态环境问题整改整治方案，实行一个问题一个方案、一套班子、一事一卡工作机制，祁连山自然保护区生态环境问题已完成整改139个、整改率92.7%，对7件涉嫌破坏环境犯罪线索移送公安机关依法侦查，依纪依规问责追究监管不严、履职不力、整改整治不到位的责任人员34名。开展全域生态文明建设专项行动。扎实推进祁连山国家公园体制试点，开展公园范围资源、人口信息调查等基础性工作。加快推进祁连山山水林田湖草生态保护修复工程，实施水环境治理、防风固沙、废旧矿山生态恢复治理等项目75个，完成投资13.1亿元。市县乡村四级河长制体系初步建立。启动祁连山地区自然资源资产负债表编制和县域生态保护红线划定工作。石羊河流域重点治理成果不断巩固和拓展，超额完成两大约束性指标，至12月25日，蔡旗断面过水量3.91亿立方米、比上年同期增加5577万立方米，民勤盆地地下水开采量8501万立方米，青土湖地下水埋深2.94米、比上年同期上升0.05米。加大防沙治沙造林力度，完成压沙9.5万亩、人工造林53.2万亩、封育33万亩、通道绿化833公里。开展凉州城区大气污染防治攻坚战，加强水、土壤、噪声等环境污染防治，全市5个国控省控地表水监测断面、县级及以上城市集中式饮用水源水质达标率100%，农村集中式饮用水源地水质达标率89%。

　　五是城乡规划建设管理不断加强。《武威市城市总体规划（2016—2030）》《武威历史文化名城保护规划（2016—2030年）》获省政府批复实施，编制完成凉州城区等8个控制性详细规划、城市综合交通等专项规划和文庙、古钟楼、鸠摩罗什寺历史文化街区保护更新规划。开展城乡规划集中宣传和专项治理，拆除违法建筑36.4万平方米。开展全域无垃圾专项行动，清理陈年垃圾70.8万吨。西郊公园、凉州植物园提升改造工程启动实施，海藏湖湿地公园加快建设。凉州城区33条背街小巷绿化、亮化、硬化治理基本完工，打通“断头路”12条，修补城区破损路面4.6万平方米。新建改建凉州城区和县城公厕31座。实施棚户区改造17368户、农村危房改造2072户。清源葡萄酒特色小镇加快建设，建成凉州蜻蜓、民勤红旗谷、天祝大红沟、古浪金水源等特色旅游示范村。凉州区入选全国休闲农业和乡村旅游示范区。创建“千村美丽”示范村省级10个、市县（区）级28个，“万村整洁”村150个。

　　六是全面深化改革纵深推进。着力推进“三去一降一补”，关闭煤矿3处、淘汰产能24万吨，关闭“地条钢”非法生产企业3户，商品住宅去化周期降至9.3个月。取消5条政府还贷二级公路收费。扎实推进“放管服”改革，取消调整市级行政审批事项21项，实施投资项目并联审批，开展“减证便民”专项行动，“双随机一公开”监管实现全覆盖。“多证合一”商事制度改革全面启动，新注册市场主体19289户。“营改增”助力供给侧结构性改革政策全面落实。农村资源变资产、资金变股金、农民变股东改革启动实施。事业单位分类改革稳步推进，机构编制总量实现零增长。大力推进知识产权战略，累计完成专利申请5268件。凉州区居家养老服务改革试点顺利推进，为符合条件的近4万名老年人提供居家养老服务。深化公交经营管理体制改革，组建武威市公交公司，淘汰“冒黑烟”公交车116辆，投入运营纯电动公交车186辆，建成智能公交系统，建设港湾式停靠站35个，更新公交候车厅112个，开通旅游专线3条。5120辆共享单车落户武威。深入推进城市执法体制改革，集中实施城市建设管理行政处罚权。深化统计管理体制改革，强化统计执法监督。国资国企改革不断深化，九条岭煤业公司改革顺利完成，中央、省属在武国有企业“三供一业”分离移交有序推进，市属国有企业法人治理结构不断完善。开展闲置土地专项清理，促进土地资源集约高效利用。清理各类政府性债务，开展不良贷款专项治理，建立风险防控机制，防范化解金融风险，政府举债融资行为逐步规范。户籍制度改革深入推进，7.34万农业人口落户城镇。

　　七是民生保障水平稳步提高。省市列18件为民办实事全面完成。城镇新增就业2.45万人，城镇登记失业率控制在3.2%。“五险合一”社保信息管理系统上线运行，公立医院综合改革深入推进，“三医联动”改革全面推开，城镇居民医保实现省内异地就医直接结算。建立全民健康档案57.3万人（份）。为60岁以上城乡居民开展免费体检。完成恶性肿瘤筛查10万人。武威肿瘤医院创建为三级甲等医院。城市低保、农村低保、农村特困供养补助完成提标。对710名有集中供养意愿完全丧失劳动能力的残疾人全部实行集中供养。新建改建农村公路1384公里，整治农村安全隐患公路340公里。新建改建村幼儿园36所、凉州城区学校3所，武威职业学院建设项目积极推进。建设乡镇及社区体育健身中心（广场）10个、村级体育场120个，成功举办市第二届运动会。开展拖欠农民工工资专项治理，为4.2万名农民工追讨欠薪5.2亿元。取缔凉州城区小燃煤锅炉，实现城区集中供热。扎实推进安全生产领域改革发展，强力排查整治安全隐患问题，安全生产形势稳定好转。加强食品药品安全监管，人民群众饮食用药安全得到保障。全面落实粮食安全责任，完成粮油购销、储备任务。开展维稳安保集中攻坚行动，排查化解矛盾纠纷，维护社会大局稳定。全面贯彻党的民族政策和宗教工作基本方针，深入开展民族团结进步创建活动，保持了民族团结、宗教和顺的良好局面。气象、人防、消防、双拥、邮政、档案、地方志、外事侨务、防震减灾、妇女儿童和慈善事业等工作取得新成绩。

　　八是政府自身建设进一步加强。开展“三比两促”、“脱贫攻坚三富三思”主题实践活动，推进“两学一做”学习教育常态化制度化。严格执行中央八项规定和省委、市委“双十条”规定精神，扎实开展“三纠三促”专项行动，严肃查处发生在群众身边的不正之风和腐败问题，开展不落实、落不实专项治理。推进依法行政和全社会尊法学法守法用法，落实法治政府建设实施方案，扎实开展“七五”普法。完善《市政府党组议事决策规则》《市政府工作规则》，规范重大事项决策程序，开展政府决策合法性审查。全面推行政务公开，行政权力网上公开透明运行实现市县乡三级全覆盖。自觉接受人大及其常委会法律监督和工作监督、政协民主监督和社会监督，认真办理人大代表议案、政协委员提案，自觉把人大、政协意见建议落到实处。强化审计监督，加大财政专项资金、扶贫资金、自然资源资产审计力度，整改问题资金7.9亿元。加强行政监察，制定实施《关于加强政府系统预防腐败工作的实施意见》，强化廉政风险防控。立案查处各类违纪违法案件691件，给予党纪政纪处分872人，移送司法机关2人，挽回经济损失675.5万元。

　　全市经济社会发展取得的成绩，是省委、省政府和市委正确领导的结果，是市人大、市政协和社会各界监督支持的结果，是全市各族人民共同努力的结果。我代表市人民政府，向全市各族人民，向各民主党派、工商联、无党派人士、人民团体，向离退休老同志和社会各界人士，向驻武部队、武警官兵、民兵预备役人员和中央、省属在武各单位，以及所有关心支持武威改革发展稳定的同志们、朋友们，表示崇高敬意和衷心感谢！

　　我们清醒认识到，我们的工作还存在许多不足，面临诸多困难和问题。深度贫困县乡村脱贫攻坚任务艰巨，稳定脱贫产业基础不牢，公共服务和基础设施薄弱。经济发展动能不足、质量不高，新兴产业发展不快，传统产业转型升级慢，外向型经济水平不高，县域经济发展滞后，项目数量少、体量小，固定资产投资下滑，工业增长疲弱，稳增长压力加大。生态环境脆弱的状况没有根本改变，保护和建设水平低，祁连山生态环境问题整改整治还不彻底，大气污染防治形势严峻。民生保障能力较弱，就业结构性矛盾突出，城乡优质教育资源配置不均衡，基层医疗服务能力不足，满足人民群众对美好生活的需要还有很多事情要做。一些改革措施落实不到位，社会治理能力有待提高，依法行政、城乡规划建设管理、土地管理使用、环境质量和安全生产监管、项目资金管理、金融风险管控、诚信建设等方面还有许多不严格、不规范的问题。一些干部作风不硬、能力不足、努力不够，不负责、怕担当、不落实、落不实的问题比较突出。我们要坚持问题导向，聚焦发展不平衡不充分的问题，保持战略定力，夯实基础、增强底气，推动经济社会持续健康发展。

2018年主要目标和重点工作

　　2018年是贯彻党的十九大精神的开局之年，是决胜全面建成小康社会、实施“十三五”规划承上启下的关键一年。市政府工作的总体思路是：全面贯彻党的十九大精神，以习近平新时代中国特色社会主义思想为指导，深入贯彻习近平总书记视察甘肃重要讲话和“八个着力”重要指示精神，坚持党对经济工作的领导，认真落实中央经济工作会议、省第十三次党代会和市委四届四次、五次全会部署，坚持稳中求进工作总基调，坚持新发展理念，按照高质量发展的要求，统筹推进“五位一体”总体布局和协调推进“四个全面”战略布局，坚持以供给侧结构性改革为主线，统筹推进稳增长、促改革、调结构、惠民生、防风险各项工作，推动经济发展质量变革、效率变革、动力变革，打好防范化解重大风险、精准脱贫、污染防治攻坚战，加强和改善民生，坚持走生态优先、绿色发展之路，努力建设经济强市、生态大市、文化旅游名市，全力打造生态美、产业优、百姓富的和谐武威。

　　经济社会发展主要预期目标是：生产总值增长6%左右，工业增加值增长5.6%，固定资产投资增长6%，一般公共预算收入增长5%，社会消费品零售总额增长8%，城镇居民人均可支配收入增长8%，农村居民人均可支配收入增长9%，居民消费价格指数涨幅控制在3%以内，城镇化率提高3个百分点，单位生产总值能耗、能源消费总量和主要污染物排放量控制在省政府下达的目标范围内。

　　实现上述目标，重点抓好九个方面工作。

　　一、打好精准脱贫攻坚战，着力补齐全面小康短板。以天祝县、古浪县和2个深度贫困镇、115个深度贫困村及特定贫困人口为重点，聚焦保证现行标准下脱贫质量和实现“两不愁三保障”，全力落实武威市和天祝县、古浪县脱贫攻坚实施方案，用有限的时间、有限的资金、解决有限的问题、完成有限的目标。把握好脱贫节奏，既防止急躁症，又防止拖延病，确保脱真贫、真脱贫。坚持扶贫对象精准、项目安排精准、资金使用精准、措施到户精准、因村派人精准、脱贫成效精准，把扶贫和扶志、扶智结合起来，全面落实“七个一批”脱贫举措，激发贫困人口内生动力，完成74个贫困村、3.18万人脱贫任务。加快发展富民产业，推进贫困片（区）设施农牧业和特色林果业提质增效，发展高原绿色有机蔬菜、旱作马铃薯、中药材、藜麦等特色产业，培育农业产业化龙头企业、专业合作社、家庭农场、种养大户等新型农业经营主体，组建国有农业公司，带动贫困户稳定脱贫，确保贫困人口人均可支配收入增速高于全市平均水平。加快易地扶贫搬迁，全面完成2016、2017年度规划搬迁任务，实施新增易地扶贫搬迁515户2166人。推进基础设施和公共服务设施建设，新建改建贫困村道路600公里，全面消除农村危房，继续实施农村饮水安全巩固提升工程，改造提升综合性文化服务中心83个、标准化村卫生室65个。落实特色产业发展专项贷款、农村基础设施和人居环境综合治理专项贷款、产业发展投资基金政策，精准谋划实施项目，助推富民产业发展和贫困片（区）人居环境改善。加强协调对接，提升东西协作和对口帮扶实效。深入开展扶贫领域作风和腐败问题专项治理，建立扶贫资金、项目常态化监管机制，确保扶贫政策和资金真正用到贫困群众身上。加强贫困村驻村工作队选派管理工作，提高帮扶能力和帮扶效果。

　　二、加快构建现代化经济体系，打造武威经济升级版。认真贯彻习近平新时代中国特色社会主义经济思想，坚定不移把发展作为第一要务，围绕推动高质量发展，做强做优实体经济，着力推进生态工业、现代农业、文化旅游产业协同发展。

　　大力发展生态工业。聚焦绿色、低碳、循环、可持续发展，以高端装备制造、新材料、新能源、生物制药、信息技术、节能环保、公共安全等领域为重点，加快发展战略性新兴产业。按照产业园区功能定位，实施园区“升级突破”行动，加快引进具有产业中高端水平的大项目、好项目，大力培育和扶持小微企业成长，支持传统产业转型升级，推进以企业为主体、市场为导向、产学研深度融合的技术创新体系建设。认真贯彻《中共中央国务院关于营造企业家健康成长环境弘扬优秀企业家精神更好发挥企业家作用的意见》，全面落实“三重”、“三一”实施方案，营造企业发展良好环境。完善领导干部帮扶企业和联系企业家制度，出台支持企业环保治理政策，帮助解决企业生产经营遇到的困难和问题，推动企业达产增效。开工建设中科院钍基熔盐堆核能系统、中节能古浪10万千瓦导热油槽式光热发电、启迪桑德环卫设备制造、静脉产业园等项目，加快普安制药5000万瓶宣肺止嗽合剂改扩建、众兴1.5万吨食用菌循环经济产业园等项目建设，北京卓立机械1万台（套）工矿民用机械设备等项目建成投产。力争新增规模以上企业30户。

　　全面实施乡村振兴战略。按照产业兴旺、生态宜居、乡风文明、治理有效、生活富裕的目标要求，制定实施乡村振兴规划。坚持以绿色有机为方向、促进农牧民增收为核心，用工业化理念推进现代农业发展。大力推行“龙头企业+专业合作组织+基地+农户”经营模式，推进设施农牧业和特色林果业提质增效，加快发展农畜产品精深加工和冷链物流，推进农业生产、加工、流通全产业链开发。发展戈壁生态农业1.5万亩，建设冷藏库容15万吨，引进和培育现代农业龙头企业5户以上。创建凉州区、民勤县、古浪县国家级现代农业示范区、产业园，加快建设民勤陶中、天祝南阳山等省级现代农业示范园，建成出口农产品生产基地90万亩。推进农村一二三产业融合发展，大力发展智慧农业、品牌农业、电商农业和观光旅游农业，打造一批田园综合体，新增“三品一标”农产品15个。主要农作物耕种收机械化率达到85%以上。在凉州区试点基础上，全面推开农村“三变”改革。扩大农村“三权”抵押贷款范围和农业主产业产品价格指数保险试点。开展乡村应绿则绿、身边增绿、见缝插绿活动。加强农村基层基础工作，健全乡村治理体系，理顺移民安置区管理服务机制。提升农民素质，培育文明乡风，创建文明乡村。

　　加快培育文化旅游支柱产业。把文化旅游业作为现代服务业发展的引擎和龙头，推动文化旅游深度融合，实施全域旅游发展规划、全域旅游发展三年行动计划、乡村旅游发展规划，落实《武威市文化旅游产业发展扶持奖励办法》，推进全域、全季、全产业链旅游。加强与巅峰智业、保利集团、省公航旅合作，成立景区管理运营公司，创建3A级景区2个、4A级景区1个。开工建设雷台景区文化旅游综合体、鸠摩罗什寺和文庙历史文化街区、民勤防沙治沙博物馆，培育建设武威创意、普康农业等文化产业基地。武威丝绸之路文化遗产博览城、民勤石羊河国家湿地公园等项目建成投运。创建全域旅游示范县、示范乡镇，建成“千合农庄”示范村10个，扶持发展一批专业旅游示范村和星级农家乐，各县区创建2—3个高标准特色旅游小镇、3—5个特色旅游村。全力推进旅游系统“厕所革命”，实施景区景点改造提升工程，加快游客集散服务中心、宾馆饭店、景区道路、房车露营地、停车场等基础设施建设。办好第二届凉州文化论坛暨中国·河西走廊第八届有机葡萄美酒节，精心策划和制作反映武威历史文化的专题片，加大宣传推介力度，提升武威文化旅游知名度。加强文化遗产保护、开发利用，深入挖掘凉州文化。力争旅游接待人数增长25%、旅游总收入增长30%。

　　加快发展外向型经济。深度融入“一带一路”，深入实施《甘肃（武威）国际陆港发展战略规划（2016—2030）》，加快陆港道路、污水处理等基础设施建设，建设武威铁路国际集装箱场站，启动运营进境木材检验检疫集中监管区、进口肉类指定查验场，稳定运营“天马号”货运班列，争取设立铁路、粮食、整车内陆进口口岸，完善甘肃（武威）国际陆港口岸功能，基本具备整体运营能力。加大招商引资力度，加速聚集生产服务业、进出口加工业、现代制造业和出口示范农业、文化旅游业项目，注重培育出口产品，形成陆港产业体系雏形。加强与丝绸之路经济带沿线国家和地区经贸合作交流，扩大面向中亚西亚、欧洲国家进出口贸易，拓展南向进出口贸易。

　　提升商贸物流业发展水平。以甘肃（武威）国际陆港为主平台，加大商贸流通龙头企业培育力度，引进建设现代化商贸物流园区、电商物流园区。加强流通基础设施建设，加快建设大型商品交易市场、农产品产地批发市场、县乡便民市场等交易平台。建成万达广场并投入运营，提升昊天农产品交易市场、金沙物流园运营水平。加大电子商务示范县建设力度，完善县乡村三级服务体系，培育网销品牌，提升农产品和特色产品网络推介、营销能力。

　　三、加强项目建设，增强发展后劲。强化项目投资稳增长、调结构、补短板的关键作用，坚持招大引强、选商选资，着力引进农产品精深加工、高端装备制造、文化旅游、生态环保等产业项目，加强招商引资项目后续服务，提高项目履约率和资金到位率，力争招商引资到位资金增长6%。紧盯国家产业政策和投资导向，加强项目谋划对接，把必要性、可行性、合规性论证作为项目决策的必经程序，落实“冬谋夏干”，扎实做好项目前期工作，提高并联审批、在线审批效率，构建亲清新型政商关系，力促项目早落地早见效。规范实施PPP项目，激发民间投资活力，加速补齐污水垃圾处理、农村道路、农田水利等基础设施短板。实施500万元及以上项目600项以上。开工建设兰（州）张（掖）铁路三四线武威段、武威民用机场、机场高速公路。北（山）仙（米寺）高速公路武威至民勤红沙梁段、凉州绕城高速和大靖至干城公路建成通车。建成农村公路1500公里，整治农村安全隐患公路500公里，提升农村公路建设、管护和运营水平。建成红崖山水库加高扩建、杂木河向陆港中心区供水工程，加快金强川灌区水源保障工程建设。

　　四、加快生态文明体制改革，建设美丽武威。深入推进全域生态文明建设专项行动，让武威的天更蓝、地更绿、水更清、空气更清新。严格落实中央环保督察反馈意见整改方案和祁连山自然保护区生态环境问题整改整治方案，全面彻底完成整改整治任务。深入推进祁连山国家公园体制试点，完成国家公园武威属区各类资源权属确认登记、管理机构重建等试点任务。高标准高质量推进祁连山山水林田湖草生态保护修复工程，完成年度规划项目33项，累计投资60%以上，植被恢复、水土保持、湿地保护、水环境状况明显改善。开展大规模国土绿化行动，实施祁连山生态恢复、防沙治沙、绿洲生态景观林建设、湿地保护恢复工程，推进农田林网建设、农村社区绿化、镇区绿化、景区绿化、产业园区绿化、通道绿化、林业有害生物防治、林业科技创新行动，加快凉州城区周边防护林带建设，完成人工造林35万亩、植树2800万株、封育36万亩。全面落实河长制，加强河流水域岸线管理，加大水污染防治力度，城镇及园区污水处理厂稳定达标运行，涉水工业企业废水达标排放，全市国控省控地表水考核断面、城市集中式饮用水水源地水质达标率100%，农村集中式饮用水水源地水质达标率90%以上。坚持全民共治、源头防治，科学、精细治理大气污染，打赢蓝天保卫战。强化土壤污染管控，加强农业面源污染防治，严防沙漠污染，推进固体废弃物安全处置和资源化利用。深入推进节水型社会建设，加快河西走廊国家级高效节水灌溉示范区项目建设，实施高效节水灌溉工程、高效农田节水技术、旱作农业技术320万亩。深化农业水价综合改革，落实最严格水资源管理制度，开展水资源消耗总量和强度双控行动，全市用水总量控制在16.46亿立方米以内，地下水开采量控制在4.84亿立方米以内。巩固石羊河流域重点治理成果，积极谋划石羊河流域后续治理项目，继续做好引黄济石精准扶贫生态移民供水工程前期工作。改革生态环境监管体制，完成省以下环保机构监测监察执法垂直管理制度改革，落实生态保护责任清单，探索建立生态保护补偿机制，建成天地一体化生态环境综合监测平台，严格生态环境执法监管，依法打击环境违法犯罪，开展跨区域联防联治。

　　五、着力改善人居环境，打造人文生态之城。严格执行《武威市城市总体规划（2016—2030）》，落实“规划为生态留足空间、为文化留足空间、为公共服务留足空间，商业开发为生态让路、为文化让路、为公共服务让路”原则，编制完成城市总体设计、风貌和色彩等专项规划，编制中心城区防洪、环卫等基础设施和教育、医疗卫生、文化体育、商业服务、无障碍设施等公共服务专项规划，完成古浪县、天祝县“多规合一”规划，加快编制县城、乡镇、园区、村庄规划，实现城镇建设用地控制性详细规划全覆盖。深入推进城乡规划专项治理，依法整治违法违规建筑，实现违法建设“零增长”。依法加强城市建设用地管理，规范土地审批和供应，坚决杜绝违法违规批地供地用地。扎实开展“厕所革命”新三年行动，新建改建农村厕所6.86万座，全面消除城乡公共旱厕。加强市政设施建设，打通凉州城区“断头路”6条，加大破损路面和人行道改造力度，改造提升供水、供热、排污、排洪管网设施，规划建设一批公共停车场、公交站点。加快公园绿地系统建设，多点布局绿地、小广场、小游园，大幅度提高凉州城区和县城绿化覆盖率。坚持全民动员、全社会参与，持续开展全域无垃圾专项行动，深化城乡环境卫生治理，推进以巷道通畅、农宅改造、村居绿化、改水改厕、硬化亮化为重点的农村人居环境综合整治，健全农村垃圾“农户分类、村组收集、乡镇转运、集中无害化处理”机制，建设美丽宜居村庄，建成省级“千村美丽”示范村15个、市县级60个，“万村整洁”村150个。推进城镇基本公共服务常住人口全覆盖，引导农业人口向城镇转移落户。加快武威市大数据云计算中心暨应用项目建设，推进信息资源共享，打造智慧城市。积极争创全国文明城市。

　　六、培育发展特色优势产业，做大做强县域经济。全面实施“1+4”县域经济发展规划，加快构建主导产业高端化、传统产业品牌化、新兴产业规模化的发展格局，走各具特色的县域经济发展之路。凉州区重点发展现代装备制造、食品加工、生物医药、家具制造、新能源、设施农牧业、现代商贸物流、历史文化游等特色产业；民勤县重点发展沙区特色种养、农畜产品精深加工、生物制药、新能源、沙漠生态文化游等特色产业；古浪县重点发展精细化工、新型建材、新能源、中药材种植加工、戈壁农业、红色文化游、历史文化游等特色产业；天祝县重点发展自然风光和民族风情游、高原绿色有机蔬菜、藜麦、食用菌、农畜产品精深加工、碳基新材料、新型建材、新能源、藏医藏药、冷链物流等特色产业。优化产业园区空间布局，加快完善供排水、污水处理、道路、供热、供气等基础设施，提升园区承载力和投资吸引力。统筹推进县域交通、水利、电力、环保、信息等基础设施建设，加强创新能力建设，加大人才、技术引进力度，推进县域经济发展。

　　七、全面深化改革，激发发展和社会活力。深化供给侧结构性改革，持续推进“三去一降一补”，继续化解过剩产能，处置“僵尸企业”，培育具有创新能力的骨干企业，降低企业制度性交易成本和用工、用能、物流、融资等经营性成本，继续清理涉企收费。加快建立多主体供应、多渠道保障、租购并举的住房制度，完善促进房地产市场平稳健康发展的长效机制，加强房地产市场调控，稳定商品房价格，加快非住宅商品房网签进度，鼓励支持城镇居民改善住房和农业转移人口进城购房，将住宅商品房库存去化周期保持在合理区间。打好防范化解重大风险攻坚战，重点防范金融风险，引导金融机构服务实体经济，推进绿色金融、普惠金融，建设融资担保体系和保险示范县，加强薄弱环节监管和重点领域风险防范处置，优化金融环境，坚决守住不发生区域性金融风险底线。推进财税体制改革，建立规范透明、标准科学、约束有力的预算制度，优化财政支出结构，优先保障扶贫和民生支出，坚决制止政府项目超概算和“钱等项目”行为。规范政府举债行为，加大化债力度，努力把政府债务控制在合理区间。深化“放管服”改革，推进协同放权，清理精简不必要的审批事项，动态调整政府部门权责清单、中介服务事项清单，强化事中事后监管，推进“互联网+行政审批”。从严管控机构编制，财政供养人员只减不增，推进事业单位改革和管理创新。坚持引进消化与自主创新并举，鼓励和支持科技创新，加快科技成果转化。深化商事制度改革，全面落实“多证合一”、电子营业执照等改革措施。推动市属国有企业完善现代企业制度，健全公司法人治理结构，加快市场化转型，围绕管资本为主加快转变国有资产监管机构职能，改革国有资本授权经营体制。推进审计监督体制改革，全面推行领导干部自然资源资产责任审计，实现对公共资金、国有资产、国有资源和领导干部经济责任审计全覆盖。深入推进城市执法体制改革，提升城市管理水平。开展第三次土地调查，推进农村房地一体确权登记。完成国有林场改革，推进集体林权制度配套改革，深化供销合作社综合改革。

　　八、着力保障和改善民生，努力让人民过上更好生活。坚持人民利益至高无上，全面推进幼有所育、学有所教、劳有所得、病有所医、老有所养、住有所居、弱有所扶。优先发展教育事业，着力解决中小学生课外负担重的问题，消除中小学超大班额158个，加强婴幼儿照护和儿童早期教育服务。适应城镇人口增长和实施全面二孩政策需求，新建改（扩）建幼儿园17所、中小学35所，学前三年教育毛入园率达到93.2%，九年义务教育巩固率保持在99.5%以上。建立普通高中生均公用经费财政拨款制度，高中阶段毛入学率达到94.2%。完善职业教育体系，创建中科院洁净新能源技术创新学院，支持武威职业学院建设甘肃省优质高职院校。深化新时代教师队伍建设改革，全面提升教师素质能力。加快健康武威建设，深化医药卫生体制改革，完善全科医生培养与使用激励机制，落实家庭医生签约服务制度，加强生育医疗保健服务，理顺疾病防控体制，培育发展中医药产业，加快建设武威重离子治疗肿瘤中心暨荣华颐养园。完善社会保障体系，实现城乡居民医保整合和异地就医即时结算，提高退休人员基本养老金、城乡居民基础养老金和基本医疗保险财政补助标准。健全孤残儿童生活保障机制。推进居家养老服务体系建设，支持社会化养老服务。坚持创业带动就业，注重解决结构性就业矛盾，城镇新增就业2.2万人，城镇登记失业率控制在4%以内。改善公共文化服务条件，建成市博物馆、影剧院、图书档案馆和10个乡镇（社区）体育健身中心、110个村级体育场，市、县体育场（馆）免费开放。大力改善城乡居民住房条件，实施棚户区改造20148户、农村危房改造693户。

　　坚持尽力而为、量力而行，着力解决人民群众最关心最直接最现实的利益问题，集中力量办好6件实事：1、改善公共服务设施，新建（改造）公园绿地10个、公共停车场23个、城市（县城）公厕74座；2、开展孕妇新生儿先天性致贫性疾病免费筛查；3、安置1000名城镇困难家庭就业困难人员就业；4、实施凉州城区天然气入户扩面行动，新增用户2.1万户；5、改善城乡居民出行条件，优化延伸新增公交线路24条；6、实施残疾人就业扶持和救助康复行动，扶持30个服务机构，培训100名服务人员，资助3200名残疾人就学就业和康复。

　　九、加强和创新社会治理，维护社会和谐稳定。加强和改进人民信访工作，畅通信访渠道，落实领导干部下访接访制度，认真排查调处矛盾纠纷，依法及时就地解决群众合理诉求。加大拖欠农民工工资问题综合治理力度，依法打击恶意欠薪行为，实行欠薪企业“黑名单”制度。深化安全领域改革发展，严格落实安全生产责任体系，加强安全生产监管信息化和应急救援能力建设，提升安全风险分级管控和隐患排查治理水平，杜绝重特大安全事故。提高防灾减灾能力，保障人民群众生命财产安全。加快社会治安防控体系建设，启动实施“雪亮工程”，严厉打击违法犯罪活动，开展交通文明提升行动。加强公共法律服务体系建设，实现贫困人口、困难群体法律援助全覆盖。做好粮食购销、流通和保供稳价工作。加强食品药品安全监管，确保让人民吃得放心、用药安全。规范物业管理，加强价格监管，解决收费任性、收费服务物非所值等问题。积极推进军民融合，密切军政军民关系。深入开展民族团结进步创建活动，依法加强民族宗教事务管理，推进天祝“两个共同”示范县建设，维护社会大局和谐稳定。

加强政府自身建设

　　做好2018年工作，加强政府自身建设是保障。各级政府要聚焦忠诚干净担当、为民务实清廉，全面加强自身建设。

　　一是加强政治建设。把学习宣传贯彻党的十九大精神作为首要政治任务，在学懂弄通做实上下功夫，用习近平新时代中国特色社会主义思想武装头脑、指导实践、推动工作。旗帜鲜明讲政治，牢固树立“四个意识”，坚决维护习近平总书记作为党中央的核心、全党的核心的地位，坚决在政治立场、政治方向、政治原则、政治道路上同以习近平同志为核心的党中央保持高度一致，坚决维护党中央权威和集中统一领导。坚定执行党的政治路线，严守政治纪律和政治规矩，严格执行新形势下党内政治生活的若干准则，始终做到党中央提倡的坚决响应、党中央决定的坚决执行、党中央禁止的坚决不做。坚决全面彻底肃清王三运流毒和影响，净化政治生态。

　　二是提升本领素质。扎实开展“不忘初心、牢记使命”主题教育，持续推进“两学一做”学习教育常态化制度化，推动各级政府更加自觉地担当使命，着力增强学习本领、政治领导本领、改革创新本领、科学发展本领、依法执政本领、群众工作本领、狠抓落实本领、驾驭风险本领，培育专业能力和专业精神，弘扬钉钉子精神和工匠精神，全面提升适应新时代新目标新任务的能力。

　　三是推进依法行政。坚持厉行法治，推进科学立法、严格执法、公正司法、全民守法。深化法治宣传教育，树立宪法法律至上、法律面前人人平等的理念，把法治思维和法治方式贯穿于政府工作全过程。健全依法决策机制，落实政府法律顾问和公职律师制度，构建决策科学、执行坚决、监督有力的权力运行机制。加强政府立法，创新执法体制，完善执法程序，推进综合执法，严格执法责任。市政府班子成员要带头学习宪法、尊崇宪法，经常对照宪法检身正己；政府系统各级干部要带头尊法学法守法用法，决不能以言代法、以权压法、逐利违法、徇私枉法。加大政务公开力度，切实保障人民群众知情权、参与权、表达权、监督权。自觉接受人大法律监督、政协民主监督、司法监督、审计监督、舆论监督、社会监督，不断改进政府工作，当好人民公仆。

　　四是持续改进作风。认真践行党的群众路线，增强群众观念和群众感情，为群众办实事、解难事，凡是群众反映强烈的问题都要严肃认真对待，凡是损害群众利益的行为都要坚决纠正。坚持群众至上、实践第一、大抓基层、从严从实导向，大兴调查研究之风，着力解决问题。弘扬求真务实之风，说实话、谋实事、出实招、求实效。严格执行《中共中央政治局贯彻落实中央八项规定的实施细则》精神和省委、市委“双十条”规定，持续纠“四风”、改作风，力戒形式主义、官僚主义，采取过硬措施整改存在问题，严防不良风气反弹回潮，推进政府系统作风明显转变。

　　五是加强廉政建设。持续推进政府系统全面从严治党，以党章为根本遵循，严格遵守党纪党规，以强化政治纪律和组织纪律带动廉洁纪律、群众纪律、工作纪律、生活纪律严起来。依纪依法管权用权，坚决反对特权思想和特权现象。严格落实党风廉政建设主体责任，强化政府系统廉政风险防控，加强对精准扶贫、重大决策、重大项目、资金拨付、政府采购、招标投标、土地管理等方面的监督，严肃查处发生在群众身边的不正之风和腐败问题，强化不敢腐的震慑，扎牢不能腐的笼子，增强不想腐的自觉，推进干部清正、政府清廉、政治清明。

　　六是提高工作效率。坚持把抓落实作为一切工作的“生命线”，严字当头、实字托底、细字绣花，着力提高各级政府执行力，制定的政策坚决落实，承诺的事项全面兑现，定了的事情马上就办，部署的工作一抓到底，不取巧、不作秀，苦干实干、务求实效。深入开展不落实、落不实专项治理，强化督查问责，推动各项工作落细落小落实。健全和落实激励机制和容错纠错机制，为敢于担当、踏实做事、不谋私利的干部撑腰鼓劲，营造想干事、能干事、干成事、不出事的良好氛围。

　　各位代表，新时代要有新气象，更要有新作为。让我们更加紧密地团结在以习近平同志为核心的党中央周围，坚持以习近平新时代中国特色社会主义思想为指导，在省委、省政府和市委的坚强领导下，不忘初心，牢记使命，振奋精神，笃实力行，为加快建设生态美、产业优、百姓富的和谐武威，决战决胜全面建成小康社会而不懈奋斗！
